# For This Good Year

**Published:** 1919-01

**Authors:** 


TEXAS MEDICAL JOURNAL

DR. F. E. DANIEL,
Founder.

Established July,
1885

MRS. F. E. DANIEL,
Publisher and Managing
Editor.

VOL. XXXIV No. 7
AUSTIN,
JANUARY, 1919.

PUBLISHED MONTHLY.
Subscription:
$1.50

The publisher is not responsible for views of contributors.

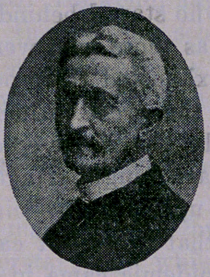

ASSISTANT EDITORS:

DR. JOHN S. TURNER, Dallas, Texas.
DR. E. B. PARSONS, Palestine, Texas.
DR. A. P. HOWARD, Houston, Texas.
DR. JOE BECTON, Greenville, Texas.
DR. M. F BLEDSOE, Port Arthur.
DR. J. H. FLORENCE, Tulsa, Oklahoma.
DR. J. H. REUSS, Cuero, Texas.

### EDITORIAL.

### For This Good Year.

This journal would like to do a little more and a little better work this year than ever before. Last year and the year before all publications, and especially medical journals, were hampered by the necessary war conditions and consequent restrictions. But the war is over, and many physicians who volunteered their services for the duration of the war, are returning to their homes to take up their lives as civilian practioners. The Texas Medical Journal, along with all the physicians and people of the country, welcomes them back. It is good to look into their faces and hear them tell of their experiences, but better than all is the satisfaction of having them home again.

We would be glad to have this journal go into the offices of all these noble men, who have so willingly sacrificed so much for their country, not so much for the money that it would bring in, but that it might be of some help to them and in return might receive help from them. Many of their experiences would make interesting and profitable reading for every member of the Journal family, and we are sure that every one of them would profit by the many helpful articles that appear from month to month.

But it is not with the soldier-physicians alone that The

Texas Medical Journal would like to do greater work than heretofore. The men who stayed behind were just as patriotic, just as self-sacrificing as those who went to the front Many of them have almost exhausted their powers of endurance in fighting here at home the great influenza plague; they have fought nobly, untiringly, the worst epidemic the country has known in years, and have fought it against many odds. The war made such a drain upon the physicians of the country that it was with difficulty that those left carried on their regular practice. When the epidemic came in all its force, many of them went for days and almost for weeks without rest or sleep. Nothing but the strongest wills could have endured what practically all the physicians in this country undertook when they so freely responded to every call for their services.

The spirit manifested by the good doctors of the United States has endeared the profession to the people more closely than ever before. The Medical Journal is glad to be classed as the friend of these heroic men here at home.

Our efforts in their behalf and for those who are coming back will be limited only by our knowledge of what is to be done and what we can do. We would be glad to have friends of the Journal write and suggest what improvements can be made in it that it may be made more interesting and more useful. Should there be more original articles? If so, what subjects would prove most interesting? Are the articles generally too long? Are they too technical? Is there enough variety to them? Should there be more clippings and extracts from other publications? Is too much or too little space given to editorials?

If in any respect the publication can be improved or made more useful by any expenditure commensurate with its patronage it will delight the publisher to make the improvement. Its only purpose is to serve the medical profession efficiently and to prove a welcome visitor wherever it goes.

---

Give to the Armenian Relief Campaign, February 3-10.

